# Personalization of Affective Models Using Classical Machine Learning: A Feasibility Study

**DOI:** 10.3390/app14041337

**Published:** 2024-02-06

**Authors:** Ali Kargarandehkordi, Matti Kaisti, Peter Washington

**Affiliations:** 1 Information and Computer Sciences Department, University of Hawai’i at Manoa, Honolulu, HI 96822, USA; 2 Department of Computing, University of Turku, 20014 Turku, Finland

**Keywords:** personalized ML, affective computing, ASD, digital phenotyping, emotion, generic

## Abstract

Our study delves into the concept of model personalization in emotion recognition, moving away from the one-size-fits-all approach. We conducted a series of experiments using the Emognition dataset, comprising physiological and video data of human subjects expressing various emotions, to investigate a personalized approach to affective computing. For the 10 individuals in the dataset with a sufficient representation of at least two ground truth emotion labels, we trained a personalized version of three classical ML models (k-nearest neighbors, random forests, and a dense neural network) on a set of 51 features extracted from each video frame. We ensured that all the frames used to train the models occurred earlier in the video than the frames used to test the model. We measured the importance of each facial feature for all the personalized models and observed differing ranked lists of the top features across the subjects, highlighting the need for model personalization. We then compared the personalized models against a generalized model trained using data from all 10 subjects. The mean F1 scores for the personalized models, specifically for the k-nearest neighbors, random forest, and dense neural network, were 90.48%, 92.66%, and 86.40%, respectively. In contrast, the mean F1 scores for the generic models, using the same ML techniques, were 88.55%, 91.78% and 80.42%, respectively, when trained on data from various human subjects and evaluated using the same test set. The personalized models outperformed the generalized models for 7 out of the 10 subjects. The PCA analyses on the remaining three subjects revealed relatively little facial configuration differences across the emotion labels within each subject, suggesting that personalized ML will fail when the variation among data points within a subject’s data is too low. This preliminary feasibility study demonstrates the potential as well as the ongoing challenges with implementing personalized models which predict highly subjective outcomes like emotion.

## 1. Introduction

Emotion recognition is vital in fields like marketing, human–robot interaction, healthcare, mental health monitoring, and security [1]. In healthcare, affective computing is crucial for understanding various neurological disorders, including sleep disorders [2], schizophrenia [3], sleep quality assessment [4], autism spectrum disorder [5,6], and Parkinson’s disease [7–9]. Emotions are also significant in identifying physiological states like fatigue, drowsiness, depression, and pain [10–12]. Emotions may be conveyed through a combination of facial expressions, vocalizations, gestures, and body movements [13–15]. This multifaceted nature of emotional expression underlines the complexity in accurately capturing and interpreting emotions through technology. Many existing works in this area rely on a one-size-fits-all emotion recognition computer vision model. However, this approach may overlook individual variations in emotional expressions and could result in less accurate assessments for certain individuals. Additionally, factors such as cultural disparities, age, and personal characteristics can influence emotional expression, posing further challenges to the effectiveness of the generic model.

Personalized models, or the creation of a separate AI model per person, offer the advantage of tailoring the emotional assessment and therapy process to each individual’s unique facial dynamics. Prior work has demonstrated that personalization can enhance the performance of emotion recognition systems [16–18]. Personalized ML techniques have the potential to unlock more precise and context-aware emotion recognition capabilities compared to the traditional paradigm of using generic models.

Here, we study the personalization of emotion recognition models using a video dataset called Emognition. As a stride towards making our models explainable, we focus in this paper on the feature extraction of interpretable facial features fed into classical ML models rather than using convolutional neural networks (CNNs) to automatically learn complex features. We train separate models per human subject and evaluate on that individual’s data, ensuring that all data in the training set occur earlier temporally than the evaluation set. Our findings demonstrate that the personalized models consistently outperform their baseline counterparts, which rely on data from other subjects within the dataset. This suggests the potential advantages of model personalization in optimizing the performance of applications requiring intricate and potentially subjective automated assessments for end-users. The implications of our study extend to domains where nuanced and individualized predictions play a crucial role in enhancing user experience and outcomes.

The code we used to train and evaluate our personalized and generalized models is available at Supplementary Materials part: https://github.com/aliknd/Personalized_Affective_Models_AMP_Paper (accessed on 2 December 2023).

## 2. Prior Work and Background

In the field of digital health, facial emotion recognition has emerged as a focal point of research, presenting an array of applications across various health domains [19,20,21]. For example, the local binary pattern (LBP) method transforms an image into a configuration of micro-patterns [22,23]. In 2009, Shan et al. [24] evaluated the effectiveness of LBP features in recognizing expressions. The results of the experiment confirmed that LBP features possess a certain level of efficiency. However, despite their usefulness, facial expression recognition methods based on LBP also suffer from challenges including low accuracy in recognition and vulnerability to interference [25]. Another feature extraction approach involves the use of Histogram of Oriented Gradients (HoG) features to recognize facial expressions. In Dahmane et al.’s study, a combination of feature extractors, such as LBP, PCA, and HoG, was used together with a SVM classifier to categorize static face images into six distinct emotions [26]. In a study conducted in 2012, Satiyan et al. [27] used Haar wavelet features along with multiscale analysis and statistical analysis to recognize facial expressions. However, the use of Haar-based facial expression recognition presents challenges, including a high rate of false recognition and the incomplete extraction of facial expression information. Another method called scale-invariant feature transform (SIFT) was used by Soyel et al. [28] in 2011 to describe face pose and achieve expression recognition through the extraction of principal component information using singular value decomposition (SVD). Nevertheless, using the SIFT-based method for expression recognition faces obstacles such as limited computational efficiency and vulnerability to dimensionality problems.

An alternative strategy for recognizing facial expressions involves deep learning. In 2006, Hinton introduced the layer-by-layer training approach to tackle the complex task of training neural networks with multiple layers [29]. As a result, robust open-source learning frameworks such as Torch, Caffe, Deep Learn Toolbox, and Cxxnet were created, supported by substantial contributions from researchers and institutions. Deep learning has the capability to approximate high-dimensional data spaces, making it well suited for learning intricate functions and extracting high-dimensional feature representations from images. While initially used for object image classification, deep learning has gradually found applications in face recognition as well [15,30,31].

In 2016, Zhang and colleagues presented a new method for recognizing facial expressions that are invariant to attitude. Their approach involved combining deep learning techniques, a principal component analysis network, and CNN. Through extensive experiments on two publicly available databases, they demonstrated substantial enhancements in their method compared to traditional techniques used for expression recognition [32]. In 2017, Zhang [33] introduced in their study an algorithm for extracting facial expressions using deep learning. They conducted an analysis of the existing approaches in this field and compared different methods. The findings revealed that deep learning techniques excel at extracting hierarchical features and leveraging them for image classification based on expressions. Consequently, these methods significantly improve the recognition accuracy when compared to conventional approaches [34–36].

Several research studies rely on facial images as a primary focus. For instance, Wells et al. [37] used transfer learning for emotion recognition with a MobileNet model. The experimental outcomes demonstrated an accuracy of 89% and an F1 score of 87%. Similarly in 2022, Ahmed et al. [38] used three pre-trained models, including MobileNet, Xception, and Inception V3, to detect ASD based on facial features. The accuracies were 95%, 94%, and 89% for MobileNet, Xception, and Inception, respectively. Another study by Akter et al. in 2021 [39] enhanced MobileNet V1 by adding layers to improve performance, achieving a classification accuracy of 90.67%.

Various studies have also analyzed the facial images of autistic children for diverse purposes. For example, in 2021, Banire et al. [40] developed a deep learning (DL) model to recognize attention from facial analysis, achieving an 88.89% accuracy and 53.1% in terms of the ACC and AUC, respectively. Washington et al. [41] conducted a study on automated emotion classification for children using a gamified approach with the GuessWhat smartphone game. The resulting extensive pediatric emotion-centric database facilitated the training of a CNN classifier, achieving a balanced accuracy of 66.9% and an F1 score of 67.4% on the entire dataset (CAFE). Notably, in a subset with at least 60% human agreement, the classifier achieved a 79.1% balanced accuracy and a 78.0% F1 score, showcasing significant improvements over previous classifiers. Kalantarian et al. [42] conducted a study evaluating the suitability of off-the-shelf emotion classifiers from Microsoft, Amazon, Google, and Sighthound for pediatric populations, specifically children with parent-reported ASD. Using the GuessWhat mobile game, 21 children with ASD engaged in social interactions, producing 2602 emotive frames for evaluation. The study revealed that while these classifiers performed well for happy emotions, their accuracy was notably poor for other emotions, indicating a need for improved training data before integrating them into AI-enabled therapeutics for autistic individuals. In the context of children with ASD, several other studies use DL and CNN for diagnosis based on facial analysis. In 2020, Beary et al. [43] introduced a DL model to classify children as normal or potentially autistic, achieving an accuracy of 94.6% using the pre-trained MobileNet model. In 2021, Nagy et al. [44] compared the accuracy of responses to six emotions (neutral, sad, disgust, anger, fear, and surprise) in normal and autistic children under non-timed and timed conditions. The results indicated that children with autism are less accurate in identifying surprise and anger compared to their neurotypical counterparts. For more comprehensive insights into emotion recognition among individuals with ASD, more expansive review papers, such as that by Rashidan et al. [45], provide detailed information.

Researchers have advocated diverse architectural modifications to CNNs, such as by incorporating the integration of attention mechanisms [46]. The visual attention mechanism enables models to concentrate on specific image regions, enhancing the overall performance. Taking inspiration from the triumph of transformer networks in natural language processing (NLP), vision transformers have been introduced. Prominent among these large transformer-based models are ViT [47], Swin [48], MobileViT [49], BiT [50], and ConvNeXt [51]. Vision transformers use attention mechanisms to encompass global context and extract intricate features from image patches. Consequently, they emerge as a promising alternative to CNNs, surmounting their challenges in feature position encoding.

Some research endeavors have harnessed transfer learning with contemporary pre-trained vision transformers for ASD diagnosis. Developments in this realm involve the utilization of VGG [52–54] and ResNet [55,56] for ASD diagnosis.

The DEAP database [57], compiled by researchers including Koelstra from various universities, serves as a valuable resource for studying human emotional states through multi-channel data. This publicly available database contains recordings of EEG signals and physiological signals (PPS) from 32 subjects. Researchers frequently use DEAP to explore and analyze the intricate aspects of human emotions. Tang et al. [58] and Yin et al. [59] are two additional studies that used a multimodal approach to emotion recognition. Both studies used deep neural networks in conjunction with the DEAP dataset, which encompasses various modalities of data such as EEG signals and physiological signals. By leveraging these multimodal data sources, Tang et al. and Yin et al. aimed to enhance the accuracy and robustness of their emotion recognition systems. The eNTERFACE’05 dataset [60] is a widely used benchmark dataset in the field of facial expression analysis and emotion recognition. The dataset consists of synchronized video recordings of facial expressions along with corresponding emotion labels. It includes expressions of basic emotions such as happiness, sadness, anger, surprise, fear, and disgust. The dataset is valuable for developing and evaluating algorithms and models for facial expression analysis, as it provides a diverse range of facial expressions and emotions in different individuals and scenarios.

Building on the foundations laid by previous research in emotion recognition, our study introduces a novel approach by focusing on the personalization of video analysis using classical ML models based on PCA features. This strategy diverges from conventional deep learning methods, presenting a distinct advantage by requiring less training data. This creates a more feasible option in scenarios with limited data availability. Moreover, PCA-based models, characterized by their reduced parameter count, are ideally suited for integration into compact health monitoring devices such as those used for digital interventions for ASD. Such an approach not only ensures computational efficiency but also enhances the interpretability of models, a key aspect of human-centered AI for healthcare.

## 3. Methods

**Figure 1.**
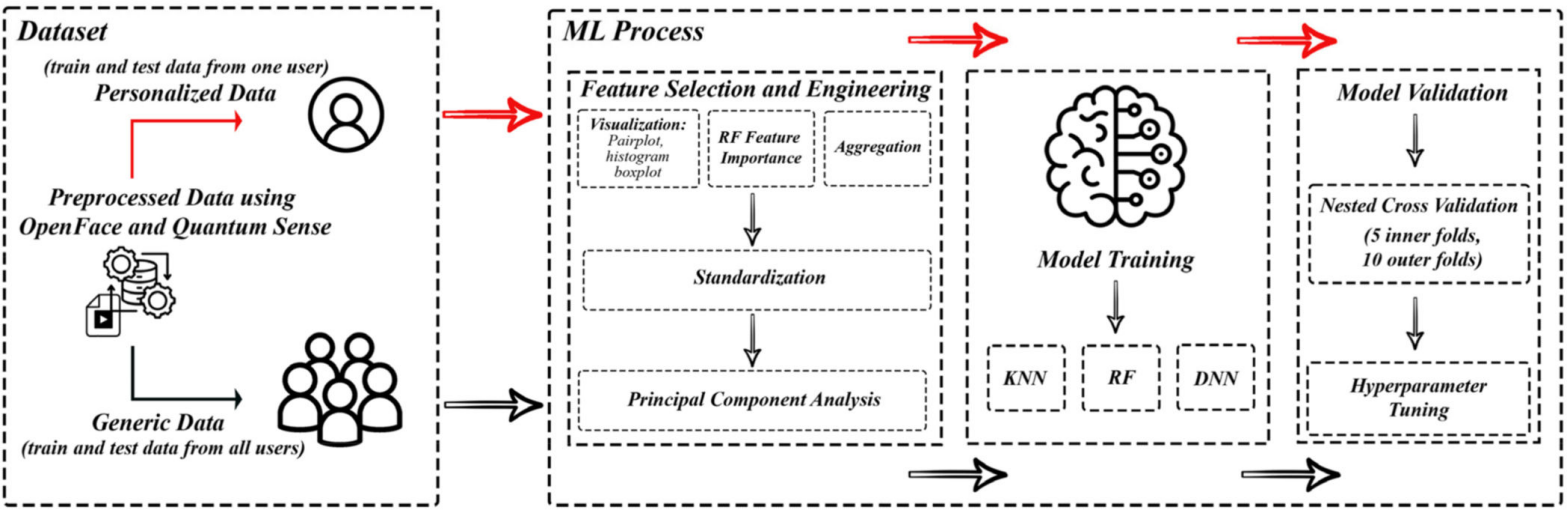
Personalized vs. generic model workflow. In addition to training a traditional one-size-fits-all model, we propose the development of a single model per individual. While we evaluate this procedure for affective computing, this paradigm can be applied to precision health more broadly.

In our study, we developed personalized emotion recognition models, as depicted in Figure 1, targeting a specific subset of subjects from the Emognition dataset.

We trained three distinct ML models: k-nearest neighbors (KNN), random forest (RF), and a dense neural network (DNN). Each of these models was applied to analyze 51 extracted facial features from each video frame, ensuring a comprehensive approach to interpreting facial expressions. The facial features were carefully chosen for their interpretability and relevance in conveying emotional states. These features include key facial landmarks, expressions, and orientations, which are pivotal in differentiating between various emotions.

### 3.1 Emognition Dataset

The Emognition dataset [63] encompasses data from 43 subjects aged between 19 and 29, including 21 females, who were exposed to emotionally stimulating film clips specifically designed to evoke nine distinct emotions (Table 1). The short film clips were chosen from databases with established reliability and validity for eliciting targeted emotions. The duration of each clip is typically short, often ranging from a few seconds to a few minutes, to maintain participant engagement and ensure a focused emotional response. These selections were made based on prior research indicating their effectiveness in evoking specific emotional responses. Facial features were automatically extracted using the OpenFace toolkit [64] (version 2.2.0, default parameters) and Quantum Sense software (Research Edition 2017, Quantum CX, Poland). The OpenFace library provides essential facial landmark points and Action Units’ values while the Quantum Sense software identifies fundamental emotions including neutral, anger, disgust, happiness, sadness, surprise (Table 1), and head pose. The ground truth for emotions was determined through participant self-selection from a pool of nine emotions: amusement, anger, awe, disgust, enthusiasm, fear, liking, sadness, and surprise, alongside assessments of valence, arousal, and motivation.

We initially considered all 43 subjects from the Emognition dataset. However, a critical challenge we faced was the uneven distribution and scarcity of emotion labels across different subjects. A significant number of subjects predominantly displayed neutral expressions, leading to an inadequate representation of diverse emotions necessary even for binary classification. To address this, we established specific selection criteria, prioritizing subjects with a balanced and sufficient number of emotion labels. This process led us to focus on 10 subjects whose data not only met our criteria for label balance but also offered a fair representation of at least two distinct emotions. This decision was pivotal in ensuring the effectiveness and accuracy of our emotion recognition analysis, albeit at the cost of reducing our sample size. The selection of these 10 subjects was imperative for maintaining the integrity of our classification task, as it provided a more reliable and representative dataset for recognizing and differentiating emotional states. Because the ≥2 emotions available in the dataset varied across these 10 subjects, we performed different classification tasks based on the label population for each emotion, ranging from binary classification to the classification of all six emotion labels.

### 3.2 Data Preprocessing and Arrangement

We observed varying elicited emotional expressions across subjects. For example, some individuals had no labels for certain emotion stimulus videos (e.g., no anger labels for anger video, no surprise label for surprise video, etc.), indicating insufficient facial expression stimulation. This disparity in label counts suggests that the threshold and manner of eliciting each emotion differ between individuals. The distribution of labels for each emotion across 9 stimulus videos and a neutral video for one demonstrative subject (no. 22) is shown in Table 1.

Notably, there were no labels for anger, surprise, and disgust emotions in subject 22′s respective video stimulus experiments. Additionally, the dataset contained only a few labels for anger and no labels for disgust at all, presenting challenges in achieving a balanced dataset for training and classification. To address this challenge, we created separate models for specific recognition tasks. To overcome the label imbalance, we collected all emotion labels specific to each discrete emotion from several video stimulus experiments into a single dataset for training each subject’s model. To mitigate the impact of imbalanced label distribution, we carefully curated emotions with a substantial number of labels for accurate and unbiased classification results. For example, for subject no. 22, we trained a model to only recognize sadness, neutral, and happiness, which were the only well-represented labels for this subject. To ensure a robust analysis, we sampled a reduced balanced subset of 1600 instances for each of the emotions for ML model training.

In contrast, for generic models, we consolidated data across all subjects, creating a “one-size-fits-all” dataset. This dataset was used to train generalized models that served as a baseline for comparison against the personalized models. The models were trained on combinations of emotions that were adequately represented across the dataset, providing a holistic view of emotion recognition across a varied population. We trained a separate model for each combination of emotions sufficiently represented by a subject (e.g., a one-size-fits-all model for happy vs. sad vs. neutral, a one-size-fits-all model for happy vs. neutral, etc.).

### 3.3. Feature Extraction and Selection

As a stride towards the development of interpretable models, we identified eye gaze, eye landmarks, pose landmarks, and Action Unit (AU) features which displayed the most predictive saliency. Eye gaze and landmarks consist of x, y, and z components. We aggregated features by calculating the mean values of each feature within each positional direction, resulting in a single combined feature value. To normalize the data, we implemented z-score standardization.

**Figure 2.**
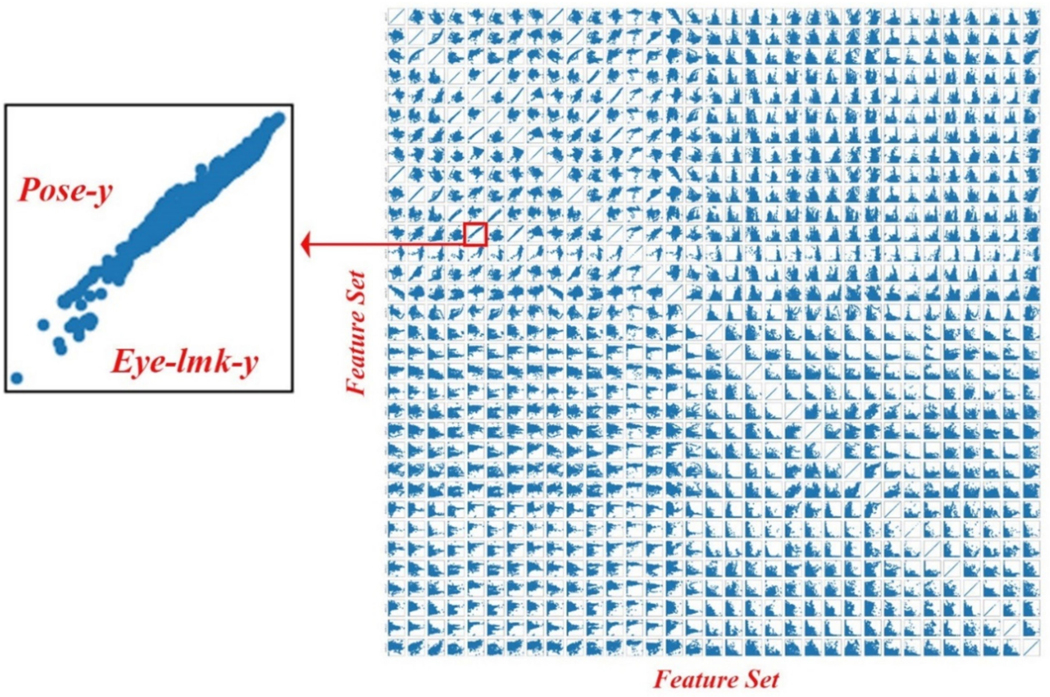
Pairwise feature correlation matrix for a demonstrative subject. We plot each feature against value against every other feature to observe correlations between features.

A pairwise feature correlation matrix for a demonstrative user (Figure 2) uncovered complex data relationships, including noticeable linear and nonlinear associations between various pairs of variables, such as the average *y*-axis coordination values of pose and eye landmarks.

**Figure 3.**
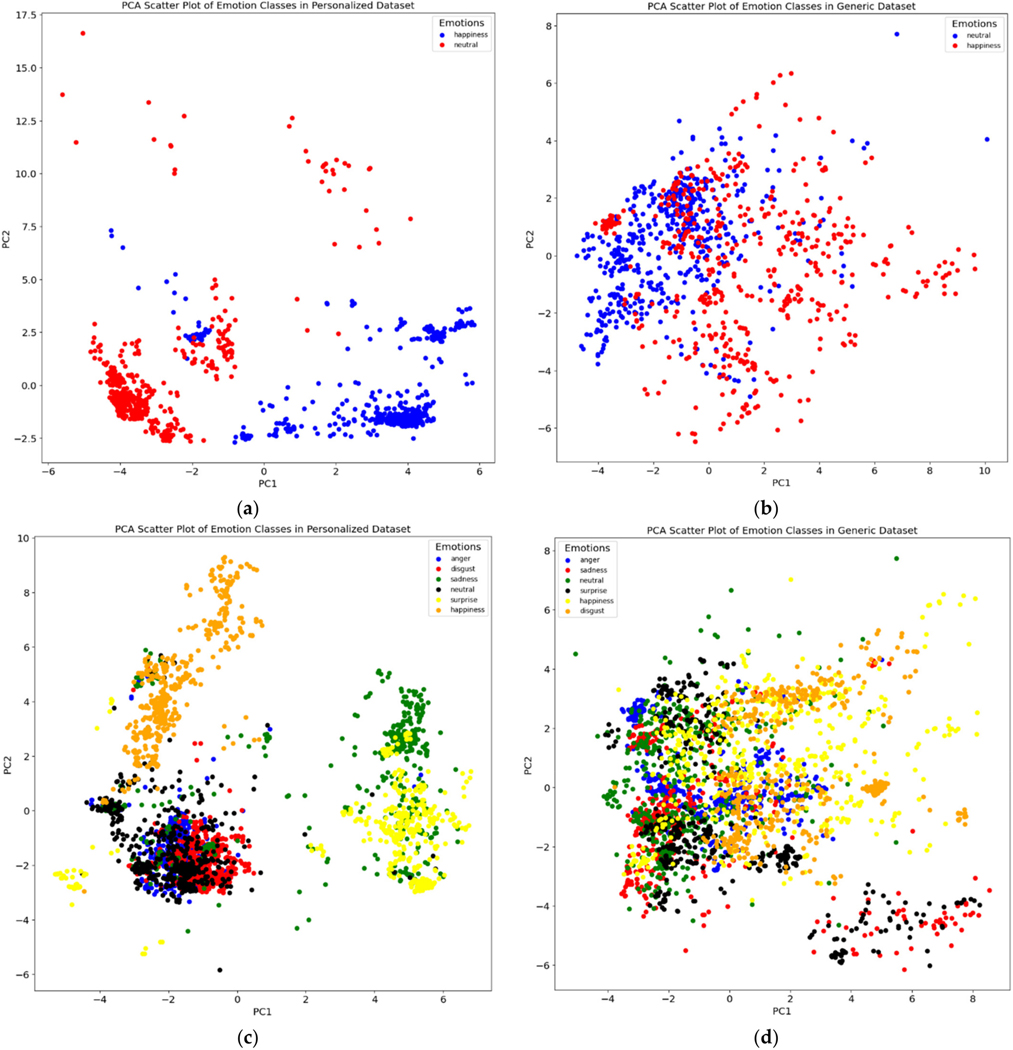
PCA visualizations of the personalized dataset for two subjects (**a**,**c**) as well as the corresponding generalized dataset (**b**,**d**).

We applied principal component analysis (PCA) to the features and color-coded each data point (i.e., video frame) based on the corresponding emotion (Figure 3 displays a demonstrative example for 2 subjects). When comparing the plots for both the personalized dataset (one individual) and the generic dataset (all ten individuals), we observed a clear difference in the separation of data points relevant to each emotion class. We observe that the personalized dataset consistently yielded a better cluster visualization compared to the generic model, suggesting the potential of the downstream ML models to result in superior performance.

### 3.4. Model Selection and Evaluation

We performed a nested cross-validation procedure to simultaneously optimize hyperparameters and assess each classifier’s performance. We performed hyperparameter tuning using grid search with an inner cross-validation of 5 folds. The best model for each classifier was selected based on evaluating different sets of hyperparameters, and its performance was evaluated on the test data using 10-fold outer cross-validation. This process ensured rigorous optimization of the classifier and complete assessment of its classification performance. We used both AUC—ROC and F1 score as our primary evaluation metrics in a one-vs-rest multiclass approach.

## 4. Results

We conducted a comprehensive evaluation of the KNN, RF, and DNN models, comparing the personalized and generalized versions of each model. In the personalized experiment, the KNN model achieved an average F1 score of 0.904, while in the generic experiment, it attained an average F1 score of 0.885. Similarly, the RF yielded average F1 scores of 0.926 and 0.917 in the personalized and generic experiments, respectively, while the MLP classifier obtained average F1 scores of 0.864 and 0.804 for the personalized and generic experiments, respectively.

We directly compare the F1 score of the personalized vs. generic models for all 10 subjects (Table 2). The personalized ML approach outperformed the general-purpose models for emotion recognition in 7 out of the 10 individuals.

**Figure 4.**
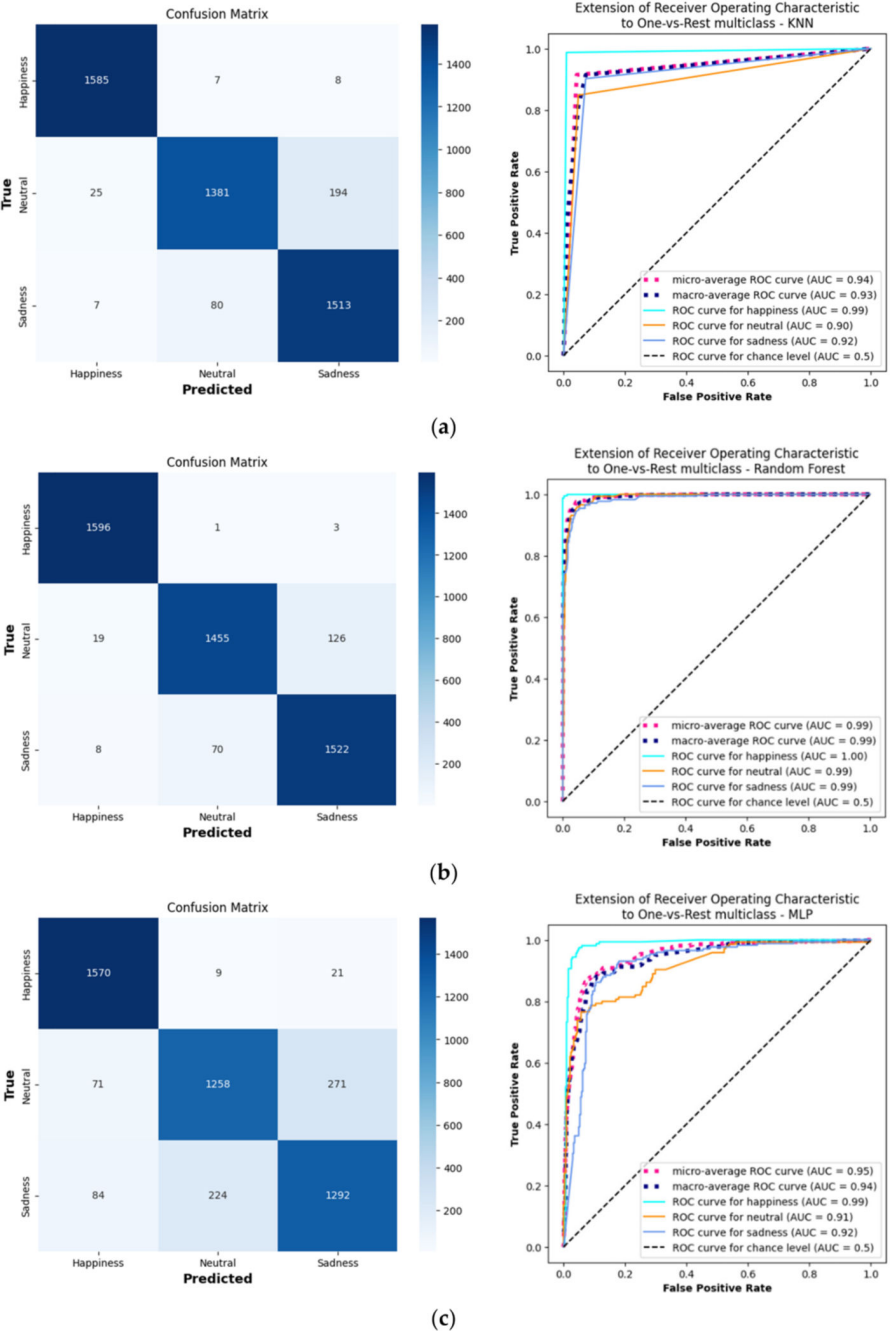
Confusion matrices and ROC curves for 3 separate models, (**a**) KNN, (**b**) RF, and (**c**) DNN, each trained and evaluated on a demonstrative subject, no. 22.

Analyzing the ROC curves and confusion matrices for a demonstrative subject (no. 22) provides insights into the personalized models’ classification capabilities (Figure 4). Retaining the best hyperparameter combinations aided in identifying the optimal settings and understanding the model’s behavior.

**Figure 5.**
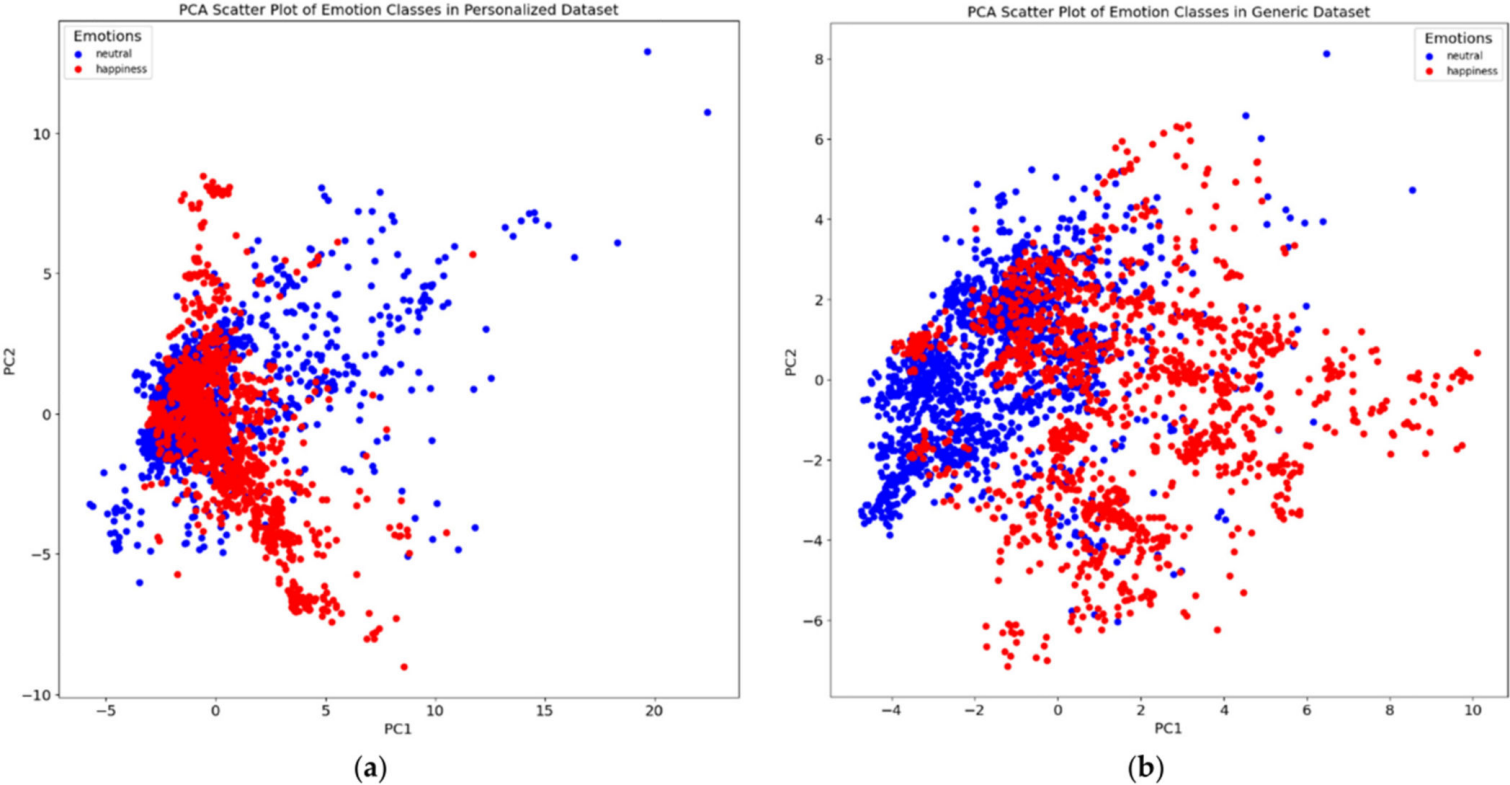
PCA plot for subject no. 39, contrasting the results in a personalized dataset (**a**) with those in a generalized dataset (**b**). In (**a**), the PCA scatter plot shows a blend of data points for “happy” and “neutral” emotional states, indicating a significant overlap and lack of clear separability in the personalized dataset. Conversely, (**b**) demonstrates a clearer distinction between the two emotional states in the generalized dataset. This comparison underlines the reason for the observed lack of performance improvement in emotion classification for this subject when using a personalized approach as opposed to a generalized one.

In certain instances, the performance of the personalized models did not surpass that of the generic models (Table 2). To investigate this discrepancy, we analyzed the PCA plots for these individuals. The PCA plots revealed that the data points representing particular emotional states did not form distinct clusters for these subjects, especially with respect to the separation of the generalized dataset containing data from all 10 subjects (e.g., Figure 5). Consequently, it is foreseeable that personalized models might encounter difficulty in accurately discerning individual emotional states compared to generic models in cases where the individual makes relatively little variation in their facial movement across emotions. The enhanced performance of generalized models in cases where subjects show little variation in emotional expressions can be attributed to their training on a more diverse range of data, which helps in better recognizing subtle emotional differences. Personalized models may struggle with these subtleties due to overfitting to specific, non-distinct features. Additionally, generalized models, being less sensitive to individual variability and noise in the data, can more effectively handle such minimal expression differences.

**Figure 6.**
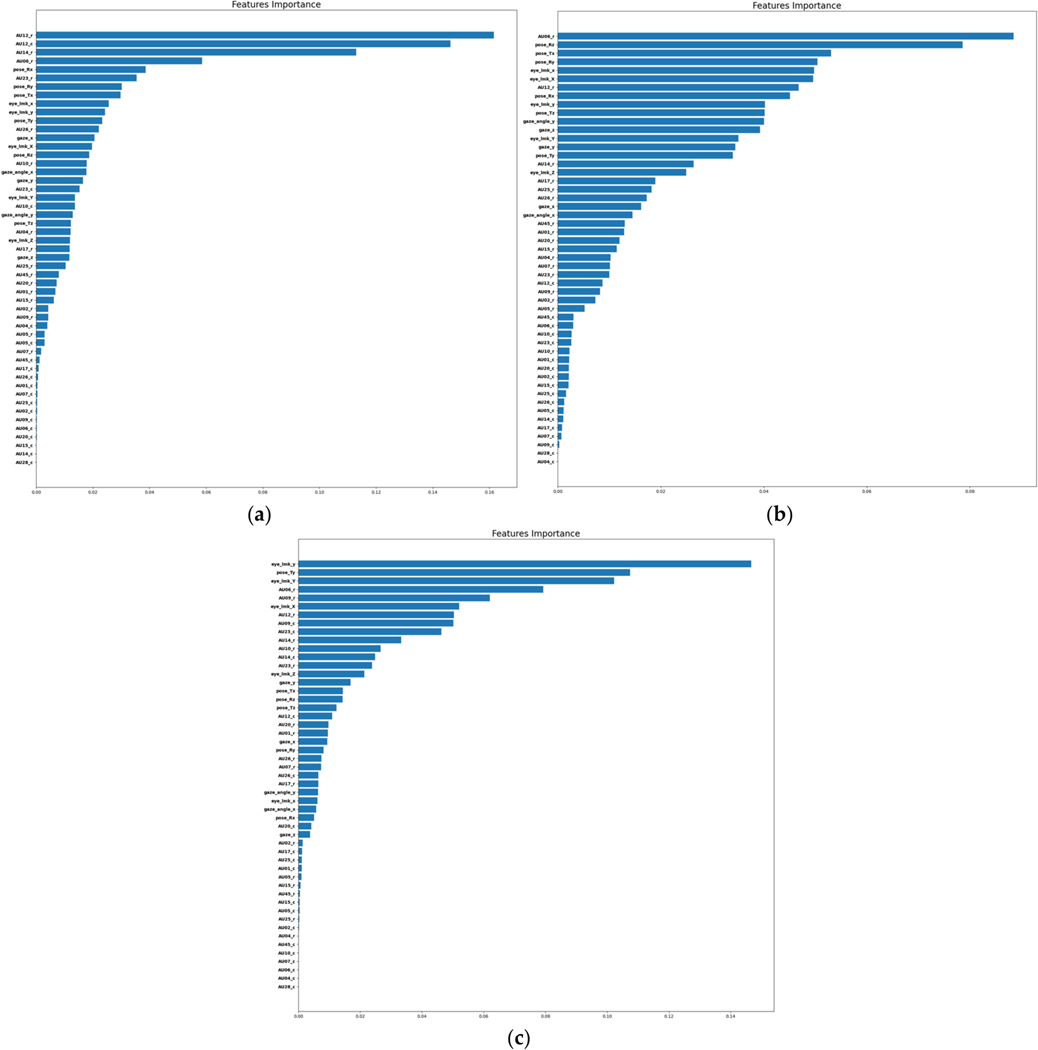
Impurity-based importance of each facial feature according to the RF model for 3 subjects’ personalized models. The model for all 3 subjects is predicting happy vs. neutral.

To explore the most salient features contributing to precise emotion classification, we computed the impurity-based importance of each feature of the RF model. This feature ranking approach inherently accounts for the correlations and complex nonlinear relationships between features. We plot the impurity-based importance of each feature in Figure 6 for a demonstrative set of three users whose ML models were all trained to predict happy vs. neutral. We observe that the top-ranked features across subjects vastly differ, further supporting the need for model personalization in affective computing.

We contextualize our findings (Table 3) by highlighting emotion recognition performances achieved in some notable prior works. We emphasize, however, that these efforts did not use the Emognition dataset that was central to our study, pre-venting us from making a direct comparison. Koelstra’s study achieved a 61.5% accuracy on the DEAP dataset using EEG and physiological signals (PPS), employing traditional signal processing techniques. Tang and Yin’s work, also on the DEAP dataset, attained an 83.5% accuracy using deep learning models that integrated EEG and PPS data, highlighting the efficacy of multimodal deep learning approaches. Nguyen’s study achieved a notable 90.85% accuracy on the eNTER-FACE’05 dataset, utilizing a combination of speech data and facial images, thereby demonstrating the potential of integrating auditory and visual cues. Zhang, using a similar dataset, achieved an accuracy of 85.97% with a focus on advanced facial expression analysis techniques. In contrast, our work with the Emognition dataset, primarily using Pose/Facial Landmarks data, yielded accuracies of 88.50% (KNN), 91.78% (RF), and 80.42% (DNN) for the generic models. Our personalized models further improved these figures to 90.48%, 92.66%, and 86.40%, respectively.

## 5. Discussion

In most cases, the personalized ML approach demonstrated a slightly stronger ability to distinguish the nuances of each subject’s emotion expressions compared to the general-purpose models. In cases where an improved performance was not observed for a subject, the PCA revealed a lack of sufficient data separation for the subject’s expressions with respect to the general-purpose models. These results support the effectiveness of the personalized ML approach in classifying emotions and highlight its potential for further advancements in the field of emotion recognition. While our examination focused on model personalization within the field of affective computing, this approach can be extended to various precision health tasks where a specific characteristic (e.g., predicting stress levels [9,15,65,66]) needs to be recurrently predicted for an individual user.

Although convolutional neural networks and vision transformers offer the possibility of better performance gains, we deliberately opted to use classical ML methods to prioritize the interpretability of our ML models. While these state-of-the-art models have demonstrated remarkable success in image recognition, their complex architecture often renders them as “black-box” models, making it challenging to interpret and understand the learned features influencing their predictions. By contrast, the automatic feature extraction we performed enabled us to inspect and comprehend the specific facial features that contribute to emotion recognition between subjects. Notably, we learned that the top facial features in the personalized models differed across subjects, highlighting the need for personalized ML.

Our study, while demonstrating promise for the personalized learning of relatively subjective tasks like affective computing, contains several limitations and can therefore only be considered as a feasibility study. We evaluated our method on only 10 subjects. While this experimental paradigm can be viewed as 10 independent N = 1 studies, we hope to expand this set of experiments in future work to more and larger datasets.

Both the evocation and understanding of emotional expressions play a crucial role in detecting certain types of developmental disorders. For instance, ASD affects almost 1 in 44 people in America [67], and it is the fastest-growing developmental disorder in the United States [68,69]. ASD is a multifaceted neuropsychiatric disorder that appears in diverse phenotypic forms. Children with autism tend to evoke emotions differently to their neurotypical peers, and they find it challenging to identify facial expressions conveyed by other individuals [70–72]. To improve the social communication of children with ASD, a variety of AI-powered mobile digital therapeutics have been developed which target emotion expression in particular [73–75]. These digital health innovations consist of smartphone apps and wearable devices that enable families to provide therapy in the comfort of their home setting with the ability to customize the intervention structure to suit their child’s needs [76–79]. For example, Superpower Glass [76,77] is an artificial-intelligence (AI)-powered digital therapeutic designed to aid children in understanding emotion evocations by conversation partners by providing real-time feedback from a facial expression recognition model. The therapeutic operates on a Google Glass connected to a smartphone and provides real-time social cues to children with ASD. “Guess What” [75] is another digital therapy encouraging, among other therapeutic behaviors, increased emotion expression using a Charades-style mobile game. Although considerable progress has been made in providing sensitive and specific emotion expression feedback to children using such digital health therapeutics, there remain several technical challenges that must be addressed to facilitate near-perfect performance.

Importantly, this study serves as a preliminary feasibility study, laying the ground-work for future work where we aim to apply these methodologies to a dataset collected from individuals with ASD. The success of our approach with the current non-ASD dataset bolsters our plan to replicate this experiment with a similar quality dataset from ASD individuals, thereby extending our study into more specialized and clinically relevant domains. An especially promising avenue of future work is the exploration of self-supervised pre-training to enhance the personalization capabilities of deep learning models. By pre-training deep learning models on large and diverse datasets using self-supervised learning, each personalized model can learn the baseline dynamics of each individual’s face without any training labels. These pre-trained models can then be fine-tuned with relatively few labeled examples. We note that this self-supervised learning paradigm would only be possible with a deep learning model rather than the classical ML approaches we present. There is a clear tradeoff between interpretability and performance.

The scalability and generalizability of personalized emotion recognition models to larger datasets and diverse populations is a crucial aspect in assessing their robustness and practical applicability. Personalized models, while highly effective in tailored scenarios, face challenges in scalability due to their inherent design for specific individuals’ emotional patterns. Generalizing these models to broader populations involves addressing variations in emotional expression across different demographics and cultures. Studies have shown that factors like cultural background and individual differences significantly impact emotional expressions and recognition [80,81]. This variability poses a challenge for personalized models when applied to a more heterogeneous group. Furthermore, scalability in terms of the dataset size can affect the model’s performance, as training on larger datasets might introduce a higher degree of variability and potential noise [82]. It is essential to consider these factors when expanding the scope of personalized models to ensure their effectiveness and reliability in diverse real-world applications.

The integration of real-time emotion recognition models in mobile health (mHealth) applications poses significant challenges, necessitating advancements in processing speed, energy efficiency, and system compatibility. Baltrušaitis et al. [83] emphasize the importance of rapid processing for real-time interaction, a critical aspect for responsive healthcare applications. Concurrently, energy efficiency, as explored by Kumar et al. [84], is paramount in mobile contexts to mitigate power consumption constraints. Furthermore, seamless integration with existing mHealth platforms, as discussed by Luxton [85], raises considerations around compatibility, data privacy, and user experience. Addressing these challenges is essential for the effective deployment of emotion recognition technologies in real-world mHealth scenarios.

## 6. Conclusions

The reliability of personalized models stems from their ability to continuously learn and adapt to the evolving emotional expression patterns of the individual user. They are often dynamic, incorporating feedback and new data over time to refine their predictions. In contrast, general models, while robust in diverse scenarios, may not offer the same level of ongoing customization and therefore might not be as reliable in capturing the subtle changes in an individual’s emotional expressions over time.

## Figures and Tables

**Table 1.** The discrepancy between the emotions evoked (actual) for one demonstrative subject vs. the emotional stimulus provided (prompt). We use the emotions evoked for our ground truth labels.

_Actual_╲^Prompt^	Amusement	Anger	Awe	Disgust	Enthusiasm	Fear	Liking	Neutral	Sadness	Surprise
Anger	0	0	0	0	0	3	0	0	0	0
Disgust	0	0	0	0	0	0	0	0	0	0
Sadness	398	213	135	227	87	111	128	1812	9	159
Neutral	6766	6752	6822	3339	7036	6993	6541	5468	7126	966
Surprise	0	37	0	0	0	116	1	0	0	0
Happiness	40	198	0	495	29	0	6	0	0	1832

**Table 2.** Overall performance (F1 score) of the models in both personalized and generic approaches on the same evaluation task. Subjects whose performance in the personalized model was lower than for the generic model are highlighted in red.

Subject	F1 Score (Personalized Models)			F1 Score (Generic Models)		

	KNN	RF	DNN	KNN	RF	DNN

No. 22	93.1%	95.3%	88.2%	86.9%	91.4%	76.7%

No. 25	89.3%	91.5%	83.4%	88.0%	92.0%	81.2%

No. 28	96.6%	97.8%	93.7%	88.5%	92.7%	82.9%

No. 29	83.6%	86.3%	78.6%	90.0%	92.0%	84.4%

No. 32	93.4%	95.1%	91.8%	92.6%	95.0%	88.0%

No. 39	87.2%	90.0%	83.8%	92.1%	94.0%	87.0%

No. 40	99.6%	99.9%	97.2%	88.3%	91.0%	78.4%

No. 42	93.2%	94.7%	89.9%	87.2%	91.6%	78.0%

No. 45	82.5%	86.3%	73.3%	82.2%	87.1%	63.9%

No. 48	86.3%	89.7%	84.1%	89.7%	91.0%	83.7%

**Table 3.** Overall performance of the generic/personalized models using in this paper and previous general model on the same task (emotion recognition).

Model	Data Type	Dataset	Metric		Value

Ours-Generic	Pose/Facial Landmarks	Emognition	F1 score	KNN	88.50%
				RF	91.78%
				DNN	80.42%
	
Ours-Personalized				KNN	90.48%
				RF	92.66%
				DNN	86.40%

## Data Availability

The data presented in this study as results are openly available in https://github.com/aliknd/Personalized_Affective_Models_AMP_Paper, accessed on 2 December 2023. The dataset used for this study can be accessed at: https://dataverse.harvard.edu/dataset.xhtml?persistentId=doi:10.7910/DVN/R9WAF4. This dataset, known as Emognition, constitutes a valuable resource for the evaluation of methodologies in emotion recognition (ER) derived from physiological responses and facial expressions. It encompasses data obtained from a cohort of 43 participants who were exposed to brief film clips meticulously designed to elicit nine distinct emotions: amusement, awe, enthusiasm, liking, surprise, anger, dis-gust, fear, and sadness. Physiological recordings were conducted using three wearable devices, enabling the capture of EEG, BVP (2x), HR, EDA, SKT, ACC (3x), and GYRO (2x) data, in conjunction with recordings of upper-body movements.

## References

[1] Kamble K; Sengupta J. A comprehensive survey on emotion recognition based on electroencephalograph (EEG) signals. Multimed. Tools Appl 2023, 82, 27269–27304.

[2] Li M; Ma C; Wu C. Facial Emotion Recognition in Sleep Deprivation: A Systematic Review and Meta-Analysis. Int. Rev. Soc. Psychol 2023, 36, 9. 10.5334/irsp.679 PMC12372770 40951795

[3] Pena-Garijo J; Lacruz M; Masanet MJ; Palop-Grau A; Plaza R; Hernandez-Merino A; Edo-Villamon S; Valllina O. Specific facial emotion recognition deficits across the course of psychosis: A comparison of individuals with low-risk, high-risk, first-episode psychosis and multi-episode schizophrenia-spectrum disorders. Psychiatry Res. 2023, 320, 115029. 10.1016/j.psychres.2022.115029 36586376

[4] Huang Y; Du J; Guo X; Li Y; Wang H; Xu J; Xu S; Wang Y; Zhang R; Xiao L. Insomnia and impacts on facial expression recognition accuracy, intensity and speed: A meta-analysis. J. Psychiatr. Res 2023, 160, 248–257. 36870234 10.1016/j.jpsychires.2023.02.001

[5] Pavez R; Diaz J; Arango-Lopez J; Ahumada D; Mendez-Sandoval C; Moreira F. Emo-mirror: A proposal to support emotion recognition in children with autism spectrum disorders. Neural Comput. Appl 2023, 35, 7913–7924. 34642548 10.1007/s00521-021-06592-5 PMC8497190

[6] Washington P; Wall DP A Review of and Roadmap for Data Science and Machine Learning for the Neuropsychiatric Phenotype of Autism. Annu. Rev. Biomed. Data Sci 2023, 6, 211–228. 37137169 10.1146/annurev-biodatasci-020722-125454 PMC11093217

[7] Belyaev M; Murugappan M; Velichko A; Korzun D. Entropy-Based Machine Learning Model for Fast Diagnosis and Monitoring of Parkinson’s Disease. Sensors 2023, 23, 8609. 37896703 10.3390/s23208609 PMC10610702

[8] Hazelton JL; Fittipaldi S; Fraile-Vazquez M; Sourty M; Legaz A; Hudson AL; Cordero IG; Salamone PC; Yoris A; Ibañez A. Thinking versus feeling: How interoception and cognition influence emotion recognition in behavioural-variant frontotemporal dementia, Alzheimer’s disease, and Parkinson’s disease. Cortex 2023, 163, 66–79. 37075507 10.1016/j.cortex.2023.02.009 PMC11177281

[9] Kargarandehkordi A; Washington P. Personalized Prediction of Stress-Induced Blood Pressure Spikes in Real Time from FitBit Data using Artificial Intelligence: A Research Protocol. medRxiv 2023.

[10] Othmani A; Sabri AQM; Aslan S; Chaieb F; Rameh H; Alfred R; Cohen D. EEG-based neural networks approaches for fatigue and drowsiness detection: A survey. Neurocomputing 2023, 557, 126709.

[11] Vehlen A; Kellner A; Normann C; Heinrichs M; Domes G. Reduced eye gaze during facial emotion recognition in chronic depression: Effects of intranasal oxytocin. J. Psychiatr. Res 2023, 159, 50–56. 36657314 10.1016/j.jpsychires.2023.01.016

[12] Dildine TC; Amir CM; Parsons J; Atlas LY How Pain-Related Facial Expressions Are Evaluated in Relation to Gender, Race, and Emotion. Affect. Sci 2023, 4, 350–369. 37293681 10.1007/s42761-023-00181-6 PMC9982800

[13] Clynes M. Sentics: The Touch of Emotions; Anchor Press: New York, NY, USA, 1977.

[14] Heraz A; Clynes M. Recognition of emotions conveyed by touch through force-sensitive screens: Observational study of humans and machine learning techniques. JMIR Ment. Health 2018, 5, e10104. 10.2196/10104 PMC6137281 30166276

[15] Kargarandehkordi A; Washington P. Computer Vision Estimation of Stress and Anxiety Using a Gamified Mobile-based Ecological Momentary Assessment and Deep Learning: Research Protocol. medRxiv 2023.

[16] Shah RV; Grennan G; Zafar-Khan M; Alim F; Dey S; Ramanathan D; Mishra J. Personalized machine learning of depressed mood using wearables. Transl. Psychiatry 2021, 11, 338. 34103481 10.1038/s41398-021-01445-0 PMC8187630

[17] Ripoli A; Sozio E; Sbrana F; Bertolino G; Pallotto C; Cardinali G; Meini S; Pieralli F; Azzini AM; Concia E. Personalized machine learning approach to predict candidemia in medical wards. Infection 2020, 48, 749–759. 32740866 10.1007/s15010-020-01488-3

[18] De Leeuw A-W; van der Zwaard S; van Baar R; Knobbe A. Personalized machine learning approach to injury monitoring in elite volleyball players. Eur. J. Sport Sci 2022, 22, 511–520. 33568023 10.1080/17461391.2021.1887369

[19] Lalitharatne TD; Tan Y; Leong F; He L; Van Zalk N; De Lusignan S; Iida F; Nanayakkara T. Facial expression rendering in medical training simulators: Current status and future directions. IEEE Access 2020, 8, 215874–215891.

[20] Picard RW Affective Computing; MIT Press: Cambridge, MA, USA, 2000.

[21] Picard RW Affective computing: Challenges. Int. J. Hum.-Comput. Stud 2003, 59, 55–64.

[22] Ahonen T; Hadid A; Pietikainen M. Face description with local binary patterns: Application to face recognition. IEEE Trans. Pattern Anal. Mach. Intell 2006, 28, 2037–2041. 17108377 10.1109/TPAMI.2006.244

[23] Ghimire D; Jeong S; Lee J; Park SH Facial expression recognition based on local region specific features and support vector machines. Multimed. Tools Appl 2017, 76, 7803–7821.

[24] Shan C; Gong S; McOwan PW Facial expression recognition based on local binary patterns: A comprehensive study. Image Vis. Comput 2009, 27, 803–816.

[25] An F; Liu Z. Facial expression recognition algorithm based on parameter adaptive initialization of CNN and LSTM. Vis. Comput 2020, 36, 483–498.

[26] Dahmane M; Meunier J. Emotion recognition using dynamic grid-based HoG features. In Proceedings of the 2011 IEEE International Conference on Automatic Face & Gesture Recognition (FG), Santa Barbara, CA, USA, 21–25 March 2011; pp. 884–888.

[27] Satiyan M; Hariharan M; Nagarajan R. Recognition of facial expression using Haar wavelet transform. J. Electr. Electron. Syst. Res. JEESR 2010, 3, 89–96.

[28] Soyel H; Demirel H. Improved SIFT matching for pose robust facial expression recognition. In Proceedings of the 2011 IEEE International Conference on Automatic Face & Gesture Recognition (FG), Santa Barbara, CA, USA, 21–25 March 2011; pp. 585–590.

[29] Hinton GE; Salakhutdinov RR Reducing the dimensionality of data with neural networks. Science 2006, 313, 504–507. 16873662 10.1126/science.1127647

[30] Banerjee A; Mutlu OC; Kline A; Surabhi S; Washington P; Wall DP Training and profiling a pediatric facial expression classifier for children on mobile devices: Machine learning study. JMIR Form. Res 2023, 7, e39917. 10.2196/39917 PMC10131663 35962462

[31] Qian Y; Kargarandehkordi A; Mutlu OC; Surabhi S; Honarmand M; Wall DP; Washington P. Computer Vision Estimation of Emotion Reaction Intensity in the Wild. arXiv 2023, arXiv:2303.10741.

[32] Zhang F; Yu Y; Mao Q; Gou J; Zhan Y. Pose-robust feature learning for facial expression recognition. Front. Comput. Sci 2016, 10, 832–844.

[33] Zhang T. Facial expression recognition based on deep learning: A survey. In Advances in Intelligent Systems and Interactive Applications, Proceedings of the 2nd International Conference on Intelligent and Interactive Systems and Applications (IISA2017), Beijing, China, 17–18 June 2017; Springer: Berlin/Heidelberg, Germany, 2017; pp. 345–352.

[34] Zhang K; Huang Y; Du Y; Wang L. Facial expression recognition based on deep evolutional spatial-temporal networks. IEEE Trans. Image Process 2017, 26, 4193–4203. 28371777 10.1109/TIP.2017.2689999

[35] Zhao X; Liang X; Liu L; Li T; Han Y; Vasconcelos N; Yan S. Peak-piloted deep network for facial expression recognition. In Proceedings of the Computer Vision–ECCV 2016: 14th European Conference, Amsterdam, The Netherlands, 11–14 October 2016; pp. 425–442.

[36] Cao C; Weng Y; Zhou S; Tong Y; Zhou K. Facewarehouse: A 3D facial expression database for visual computing. IEEE Trans. Vis. Comput. Graph 2013, 20, 413–425. 10.1109/TVCG.2013.249 24434222

[37] Wells LJ; Gillespie SM; Rotshtein P. Identification of emotional facial expressions: Effects of expression, intensity, and sex on eye gaze. PLoS ONE 2016, 11, e0168307. 10.1371/journal.pone.0168307 PMC5152920 27942030

[38] Ahmed ZA; Aldhyani TH; Jadhav ME; Alzahrani MY; Alzahrani ME; Althobaiti MM; Alassery F; Alshaflut A; Alzahrani NM; Al-Madani AM Facial features detection system to identify children with autism spectrum disorder: Deep learning models. Comput. Math. Methods Med 2022, 2022, 3941049. 10.1155/2022/3941049 PMC9001065 35419082

[39] Akter T; Ali MH; Khan MI; Satu MS; Uddin MJ; Alyami SA; Ali S; Azad A; Moni MA Improved transfer-learning-based facial recognition framework to detect autistic children at an early stage. Brain Sci. 2021, 11, 734. 34073085 10.3390/brainsci11060734 PMC8230000

[40] Banire B; Al Thani D; Qaraqe M; Mansoor B. Face-based attention recognition model for children with autism spectrum disorder. J. Healthc. Inform. Res 2021, 5, 420–445. 35415454 10.1007/s41666-021-00101-y PMC8982782

[41] Washington P; Kalantarian H; Kent J; Husic A; Kline A; Leblanc E; Hou C; Mutlu C; Dunlap K; Penev Y. Improved Digital Therapy for Developmental Pediatrics Using Domain-Specific Artificial Intelligence: Machine Learning Study. JMIR Pediatr Parent 2022, 5, e26760. 10.2196/26760 PMC9034430 35394438

[42] Kalantarian H; Jedoui K; Dunlap K; Schwartz J; Washington P; Husic A; Tariq Q; Ning M; Kline A; Wall DP The performance of emotion classifiers for children with parent-reported autism: Quantitative feasibility study. JMIR Ment. Health 2020, 7, e13174. 10.2196/13174 PMC7160704 32234701

[43] Beary M; Hadsell A; Messersmith R; Hosseini M-P Diagnosis of autism in children using facial analysis and deep learning. arXiv 2020, arXiv:2008.02890. 10.3389/fncom.2021.789998 PMC8811190 35126078

[44] Nagy E; Prentice L; Wakeling T. Atypical facial emotion recognition in children with autism spectrum disorders: Exploratory analysis on the role of task demands. Perception 2021, 50, 819–833. 34428977 10.1177/03010066211038154 PMC8438782

[45] Rashidan MA; Na’im Sidek S; Yusof HM; Khalid M; Dzulkarnain AAA; Ghazali AS; Zabidi SAM; Sidique FAA Technology-assisted emotion recognition for autism spectrum disorder (ASD) children: A systematic literature review. IEEE Access 2021, 9, 33638–33653.

[46] Vaswani A; Shazeer N; Parmar N; Uszkoreit J; Jones L; Gomez AN; Kaiser Ł; Polosukhin I. Attention is all you need. Adv. Neural Inf. Process. Syst 2017, 30, 6000–6010.

[47] Dosovitskiy A; Beyer L; Kolesnikov A; Weissenborn D; Zhai X; Unterthiner T; Dehghani M; Minderer M; Heigold G; Gelly S. An image is worth 16×16 words: Transformers for image recognition at scale. arXiv 2020, arXiv:2010.11929.

[48] Liu Z; Lin Y; Cao Y; Hu H; Wei Y; Zhang Z; Lin S; Guo B. Swin transformer: Hierarchical vision transformer using shifted windows. In Proceedings of the IEEE/CVF International Conference on Computer Vision, Montreal, QC, Canada, 10–17 October 2021; pp. 10012–10022.

[49] Mehta S; Rastegari M. Mobilevit: Light-weight, general-purpose, and mobile-friendly vision transformer. arXiv 2021, arXiv:2110.02178.

[50] Kolesnikov A; Beyer L; Zhai X; Puigcerver J; Yung J; Gelly S; Houlsby N. Big transfer (bit): General visual representation learning. In Proceedings of the Computer Vision–ECCV 2020: 16th European Conference, Glasgow, UK, 23–28 August 2020; pp. 491–507.

[51] Liu Z; Mao H; Wu C-Y; Feichtenhofer C; Darrell T; Xie S. A convnet for the 2020s. In Proceedings of the IEEE/CVF Conference on Computer Vision and Pattern Recognition, New Orleans, LA, USA, 18–24 June 2022; pp. 11976–11986.

[52] Sharif H; Khan RA A novel machine learning based framework for detection of autism spectrum disorder (ASD). Appl. Artif. Intell 2022, 36, 2004655.

[53] Ahmed MR; Zhang Y; Liu Y; Liao H. Single volume image generator and deep learning-based ASD classification. IEEE J. Biomed. Health Inform 2020, 24, 3044–3054. 32750917 10.1109/JBHI.2020.2998603

[54] Yang M; Cao M; Chen Y; Chen Y; Fan G; Li C; Wang J; Liu T. Large-scale brain functional network integration for discrimination of autism using a 3-D deep learning model. Front. Hum. Neurosci 2021, 15, 687288. 10.3389/fnhum.2021.687288 PMC8206477 34149385

[55] Gao J; Chen M; Li Y; Gao Y; Li Y; Cai S; Wang J. Multisite autism spectrum disorder classification using convolutional neural network classifier and individual morphological brain networks. Front. Neurosci 2021, 14, 629630. 10.3389/fnins.2020.629630 PMC7877487 33584183

[56] Tang M; Kumar P; Chen H; Shrivastava A. Deep multimodal learning for the diagnosis of autism spectrum disorder. J. Imaging 2020, 6, 47. 34460593 10.3390/jimaging6060047 PMC8321065

[57] Koelstra S; Muhl C; Soleymani M; Lee J-S; Yazdani A; Ebrahimi T; Pun T; Nijholt A; Patras I. Deap: A database for emotion analysis; using physiological signals. IEEE Trans. Affect. Comput 2011, 3, 18–31.

[58] Tang H; Liu W; Zheng W-L; Lu B-L Multimodal emotion recognition using deep neural networks. In Proceedings of the Neural Information Processing: 24th International Conference, ICONIP 2017, Guangzhou, China, 14–18 November 2017; pp. 811–819.

[59] Yin Z; Zhao M; Wang Y; Yang J; Zhang J. Recognition of emotions using multimodal physiological signals and an ensemble deep learning model. Comput. Methods Programs Biomed 2017, 140, 93–110. 28254094 10.1016/j.cmpb.2016.12.005

[60] Martin O; Kotsia I; Macq B; Pitas I. The eNTERFACE’05 audio-visual emotion database. In Proceedings of the 22nd International Conference on Data Engineering Workshops (ICDEW’06), Atlanta, GA, USA, 3–7 April 2006; p. 8.

[61] Zhang S; Zhang S; Huang T; Gao W; Tian Q. Learning affective features with a hybrid deep model for audio–visual emotion recognition. IEEE Trans. Circuits Syst. Video Technol. 2017, 28, 3030–3043.

[62] Nguyen D; Nguyen K; Sridharan S; Dean D; Fookes C. Deep spatio-temporal feature fusion with compact bilinear pooling for multimodal emotion recognition. Comput. Vis. Image Underst 2018, 174, 33–42.

[63] Saganowski S; Komoszyńska J; Behnke M; Perz B; Kunc D; Klich B; Kaczmarek ŁD; Kazienko P. Emognition dataset: Emotion recognition with self-reports, facial expressions, and physiology using wearables. Sci. Data 2022, 9, 158. 35393434 10.1038/s41597-022-01262-0 PMC8989970

[64] Baltrusaitis T; Zadeh A; Lim YC; Morency L-P Openface 2.0: Facial behavior analysis toolkit. In Proceedings of the 2018 13th IEEE international conference on automatic face & gesture recognition (FG 2018), Xi’an, China, 15–19 May 2018; pp. 59–66.

[65] Parousidou V-C Personalized Machine Learning Benchmarking for Stress Detection. Master’s Thesis, Aristotle University of Thessaloniki, Thessaloniki, Greece, 2023.

[66] Tazarv A; Labbaf S; Reich SM; Dutt N; Rahmani AM; Levorato M. Personalized stress monitoring using wearable sensors in everyday settings. In Proceedings of the 2021 43rd Annual International Conference of the IEEE Engineering in Medicine & Biology Society (EMBC), virtually, 1–5 November 2021; pp. 7332–7335. 10.1109/EMBC46164.2021.9630224 34892791

[67] Christensen DL; Van Naarden Braun K; Baio J; Bilder D; Charles J; Constantino JN; Daniels J; Durkin MS; Fitzgerald RT; Kurzius-Spencer M. Prevalence and characteristics of autism spectrum disorder among children aged 8 years—Autism and developmental disabilities monitoring network, 11 sites, United States, 2012. MMWR Surveill. Summ 2018, 65, 1–23. 27031587 10.15585/mmwr.ss6503a1 PMC7909709

[68] Ardhanareeswaran K; Volkmar F. Introduction. Focus: Autism spectrum disorders. Yale J. Biol. Med 2015, 88, 3–4. 25902571 PMC4345536

[69] Gordon-Lipkin E; Foster J; Peacock G. Whittling down the wait time: Exploring models to minimize the delay from initial concern to diagnosis and treatment of autism spectrum disorder. Pediatr. Clin 2016, 63, 851–859. 10.1016/j.pcl.2016.06.007 PMC5583718 27565363

[70] Manfredonia J; Bangerter A; Manyakov NV; Ness S; Lewin D; Skalkin A; Boice M; Goodwin MS; Dawson G; Hendren R. Automatic recognition of posed facial expression of emotion in individuals with autism spectrum disorder. J. Autism Dev. Disord 2019, 49, 279–293. 30298462 10.1007/s10803-018-3757-9

[71] Nag A; Haber N; Voss C; Tamura S; Daniels J; Ma J; Chiang B; Ramachandran S; Schwartz J; Winograd T. Toward continuous social phenotyping: Analyzing gaze patterns in an emotion recognition task for children with autism through wearable smart glasses. J. Med. Internet Res 2020, 22, e13810. 10.2196/13810 PMC7203617 32319961

[72] Lakkapragada A; Kline A; Mutlu OC; Paskov K; Chrisman B; Stockham N; Washington P; Wall DP The classification of abnormal hand movement to aid in autism detection: Machine learning study. JMIR Biomed. Eng 2022, 7, e33771.

[73] Washington P; Voss C; Haber N; Tanaka S; Daniels J; Feinstein C; Winograd T; Wall D. A wearable social interaction aid for children with autism. In Proceedings of the 2016 CHI Conference Extended Abstracts on Human Factors in Computing Systems, San Jose, CA, USA, 7–12 May 2016; pp. 2348–2354.

[74] Voss C; Haber N; Wall DP The potential for machine learning–based wearables to improve socialization in teenagers and adults with autism spectrum disorder—Reply. JAMA Pediatr. 2019, 173, 1106. 10.1001/jamapediatrics.2019.2969 31498377

[75] Kalantarian H; Washington P; Schwartz J; Daniels J; Haber N; Wall DP Guess What? Towards Understanding Autism from Structured Video Using Facial Affect. J. Healthc. Inform. Res 2019, 3, 43–66. 33313475 10.1007/s41666-018-0034-9 PMC7730314

[76] Kline A; Voss C; Washington P; Haber N; Schwartz H; Tariq Q; Winograd T; Feinstein C; Wall DP Superpower glass. GetMobile Mob. Comput. Commun 2019, 23, 35–38.

[77] Haber N; Voss C; Wall D. Making emotions transparent: Google Glass helps autistic kids understand facial expressions through augmented-reaiity therapy. IEEE Spectr. 2020, 57, 46–52.

[78] Washington P; Voss C; Kline A; Haber N; Daniels J; Fazel A; De T; Feinstein C; Winograd T; Wall D. SuperpowerGlass: A wearable aid for the at-home therapy of children with autism. Proc. ACM Interact. Mob. Wearable Ubiquitous Technol 2017, 1, 1–22.

[79] Voss C; Washington P; Haber N; Kline A; Daniels J; Fazel A; De T; McCarthy B; Feinstein C; Winograd T. Superpower glass: Delivering unobtrusive real-time social cues in wearable systems. In Proceedings of the 2016 ACM International Joint Conference on Pervasive and Ubiquitous Computing: Adjunct, Heidelberg, Germany, 12–16 September 2016; pp. 1218–1226.

[80] Elfenbein HA; Ambady N. On the universality and cultural specificity of emotion recognition: A meta-analysis. Psychol. Bull 2002, 128, 203. 11931516 10.1037/0033-2909.128.2.203

[81] Jack RE; Garrod OG; Yu H; Caldara R; Schyns PG Facial expressions of emotion are not culturally universal. Proc. Natl. Acad. Sci. USA 2012, 109, 7241–7244. 22509011 10.1073/pnas.1200155109 PMC3358835

[82] Zeng Z; Pantic M; Roisman GI; Huang TS A survey of affect recognition methods: Audio, visual and spontaneous expressions. In Proceedings of the 9th International Conference on Multimodal Interfaces, Nagoya, Japan, 12–15 November 2007; pp. 126–133.

[83] Baltrušaitis T; Robinson P; Morency L-P Openface: An open source facial behavior analysis toolkit. In Proceedings of the 2016 IEEE Winter Conference on Applications of Computer Vision (WACV), Lake Placid, NY, USA, 7–10 March 2016; pp. 1–10.

[84] Kumar M; Zhang X; Liu L; Wang Y; Shi W. Energy-efficient machine learning on the edges. In Proceedings of the 2020 IEEE International Parallel and Distributed Processing Symposium Workshops (IPDPSW), New Orleans, LA, USA, 18–22 May 2020; pp. 912–921.

[85] Luxton DD Artificial Intelligence in Behavioral and Mental Health Care; Elsevier: Amsterdam, The Netherlands, 2015.

[86] Mohammad SM Ethics sheet for automatic emotion recognition and sentiment analysis. Comput. Linguist 2022, 48, 239–278.

[87] Boyd KL; Andalibi N. Automated emotion recognition in the workplace: How proposed technologies reveal potential futures of work. Proc. ACM Hum.-Comput. Interact 2023, 7, 1–37.

